# Revealing microhabitat requirements of an endangered specialist lizard with LiDAR

**DOI:** 10.1038/s41598-022-08524-2

**Published:** 2022-03-25

**Authors:** Holly S. Bradley, Michael D. Craig, Adam T. Cross, Sean Tomlinson, Michael J. Bamford, Philip W. Bateman

**Affiliations:** 1 ARC Centre for Mine Site Restoration, School of Molecular and Life Sciences, Curtin University, Kent Street, Bentley, Perth, WA 6102 Australia; 2 School of Biological Sciences, University of Western Australia, Crawley, WA 6009 Australia; 3 School of Veterinary and Life Sciences, Murdoch University, Murdoch, WA 6150 Australia; 4 EcoHealth Network (http://ecohealthglobal.org), 1330 Beacon St, Suite 355a, Brookline, MA 02446 USA; 5 School of Molecular and Life Sciences, Curtin University, Kent Street, Bentley, Perth, WA 6102 Australia; 6 Kings Park Science, Department of Biodiversity, Conservation and Attractions, Kattij Close, Kings Park, WA 6005 Australia; 7 Present Address: School of Biological Sciences, University of Adelaide, North Terrace, Adelaide, SA 5000 Australia; 8 Bamford Consulting Ecologists, Plover Way, Kingsley, WA 6026 Australia; 9 Behavioural Ecology Laboratory, School of Molecular and Life Sciences, Curtin University, Kent Street, Bentley, Perth, WA 6102 Australia

**Keywords:** Conservation biology, Ecology, Zoology, Herpetology

## Abstract

A central principle of threatened species management is the requirement for detailed understanding of species habitat requirements. Difficult terrain or cryptic behaviour can, however, make the study of habitat or microhabitat requirements difficult, calling for innovative data collection techniques. We used high-resolution terrestrial LiDAR imaging to develop three-dimensional models of log piles, quantifying the structural characteristics linked with occupancy of an endangered cryptic reptile, the western spiny-tailed skink (*Egernia stokesii badia*). Inhabited log piles were generally taller with smaller entrance hollows and a wider main log, had more high-hanging branches, fewer low-hanging branches, more mid- and understorey cover, and lower maximum canopy height. Significant characteristics linked with occupancy were longer log piles, an average of three logs, less canopy cover, and the presence of overhanging vegetation, likely relating to colony segregation, thermoregulatory requirements, and foraging opportunities. In addition to optimising translocation site selection, understanding microhabitat specificity of *E. s. badia* will help inform a range of management objectives, such as targeted monitoring and invasive predator control. There are also diverse opportunities for the application of this technology to a wide variety of future ecological studies and wildlife management initiatives pertaining to a range of cryptic, understudied taxa.

## Introduction

Contemporary wildlife extinctions are occurring at a rate thousands of times greater than background species loss^1^ and are predicted to increase as a result of anthropogenic threats increasing in range and intensity commensurate with increasing human population pressures^2^. As a result, threatened species recovery is a major conservation focus around the world^3^. Fundamental to a broad range of species recovery and wildlife management initiatives is understanding the two main factors that influence habitat use: habitat availability and habitat choice^4^. The first restricts the distribution of species only through the quantity of options within the surrounding environment, while the latter is linked to specific adaptations to particular habitats, regardless of what broader spectrum of habitat is available^4^. Species limited by habitat choice generally exhibit narrow niche breadth, meaning that they are constrained by the physical conditions under which they can survive and reproduce^5,6^. The more specialised the habitat/microhabitat requirements of a species, the more targeted habitat selection is necessary for successful species recovery.

Distribution maps based on historical sightings and species distribution models are often a useful starting point for developing a broad sense of habitat requirements, due to their ability to identify patterns at a coarse scale^7,8^. However, the dynamic nature of ecological systems means that species-specific microhabitat and habitat suitability assessments may be critical for finer understanding of a species’ requirements^9^, and such data are difficult to obtain from conventional distribution models^10,11^. For example, in developing habitat selection protocols for narrow-range plants, Tomlinson et al.^10^, noted that the resolution of many distribution maps were unable to identify the specific microhabitats required. For animals, these can be influenced by numerous factors including refuge from predators^12^, thermoregulatory requirements^13–15^, dispersal ability^16^, and suitability for camouflage^17,18^. However, in some cases, the nature of the environment (such as dense jungle and deep ocean), or the shy or cryptic nature of the target species can make habitat assessments by direct observation difficult^19^. Such challenges call for innovative approaches, such as the use of acoustic monitoring^19–22^, camera trapping^23–25^, and tracking tunnels^26–28^. The choice of technology is species-specific, set by the limitations of the species’ cryptic nature^29^.

The microhabitat requirements of many animals are relatively subtle, and relate to small differences in localised habitat structure^30,31^, especially for relatively sessile species^32,33^ A novel option for assessment of localised habitat structure is LiDAR (light detection and ranging^34^), a non-destructive tool that rapidly and precisely digitises an object or site into a three-dimensional (3D) point cloud^34,35^. LiDAR has been applied to the broad-scale assessment of numerous fauna habitats, including forests^36–38^, tidal flats^39^, subtidal coastal zones^40^, and rivers^41,42^, but most of these have been at relatively large scales at square metre resolutions. At a smaller scale, terrestrial LiDAR allows for detailed scanning of microhabitat structure without obstruction from overhanging canopy or vegetation. We propose that the ultra-high-resolution (10 mm) precise characterisation of the physical environment provided by terrestrial LiDAR scanning provides a unique opportunity to gain an in-depth understanding of log pile microhabitat requirements for fauna of conservation concern.

Coarse woody debris, such as fallen log piles, are often critical habitat features for threatened fauna such as numbats (*Myrmecobius fasciatus*)^43^, chuditch (*Dasyurus geoffroii*)^44^, and the western spiny-tailed skink (*Egernia stokesii badia*)^45^. However, not all log pile sites are equally suitable for species habitation, and species-specific preferences for features such as log diameter, canopy cover and presence of adjacent trees can influence site suitability^46^. Here, to our knowledge, we report the first study using ultra high-resolution terrestrial LiDAR to quantify the microhabitat characteristics of fallen log piles, using this approach to estimate the suitability of log piles for an endangered cryptic reptile subspecies (western spiny-tailed skinks). We aimed to determine if the skinks exhibited a high selectivity for certain structural features of log pile habitat, such as structural complexity with multiple logs providing potential refuge options, or associated features such as degree of vegetation cover (e.g., canopy gaps for basking) through comparison of log piles known to be inhabited and uninhabited by the species. Such detailed analysis of log pile characteristics and understanding skink microhabitat specificity will provide crucial information in the design of future habitat improvement for management efforts, highlighting the applicability of the technology for the assessment of other complex microhabitat structures, potentially including the specific structures of specialised roosting habitats in some species of bats^47,48^ or nesting hollows or sites of endangered parrots^49,50^ to better understand sites for protection, translocation, or replication in restoration and other threatened species management.

## Methods

### Study species

*Egernia s. badia* are a large skink, with both sexes reaching a mature size over 170 mm snout-vent length (SVL)^51^. Females bear live young, with some *E. stokesii* subspecies producing litters of up to eight individuals at a time^52^. The family groups are social and live together in groups comprised of different sexes and age classes. These social aggregations occupy the same large fallen log pile^45^ over years, inhabiting the hollows and crevices in the wood. The juvenile skinks also take over five years to reach mature size, with many skinks remaining in the same social group as their parents even after reaching maturity^51^. Beyond this basic information, the ecology of *E. s. badia* is largely unknown, compared to comparatively well-studied subspecies of *E. stokesii* occurring in states outside of Western Australia.

*Egernia s. badia* are at risk of extinction and are recognised under both Australian Federal legislation (Endangered; *Environment Protection and Biodiversity Conservation Act 1999*) and Western Australian state legislation (Schedule 1; *Biodiversity Conservation Act 2016*). One of the major threats identified for this subspecies is habitat loss and modification through mining and grazing; the translocation of specific populations threatened by local extinction is a recommended recovery option^45^. Although there have been no successful translocations of this subspecies published in the scientific literature, there are anecdotal reports of failed attempts which may indicate that the skink has specific log pile requirements to ensure establishment and persistence.

### Study area

**Figure 1:**
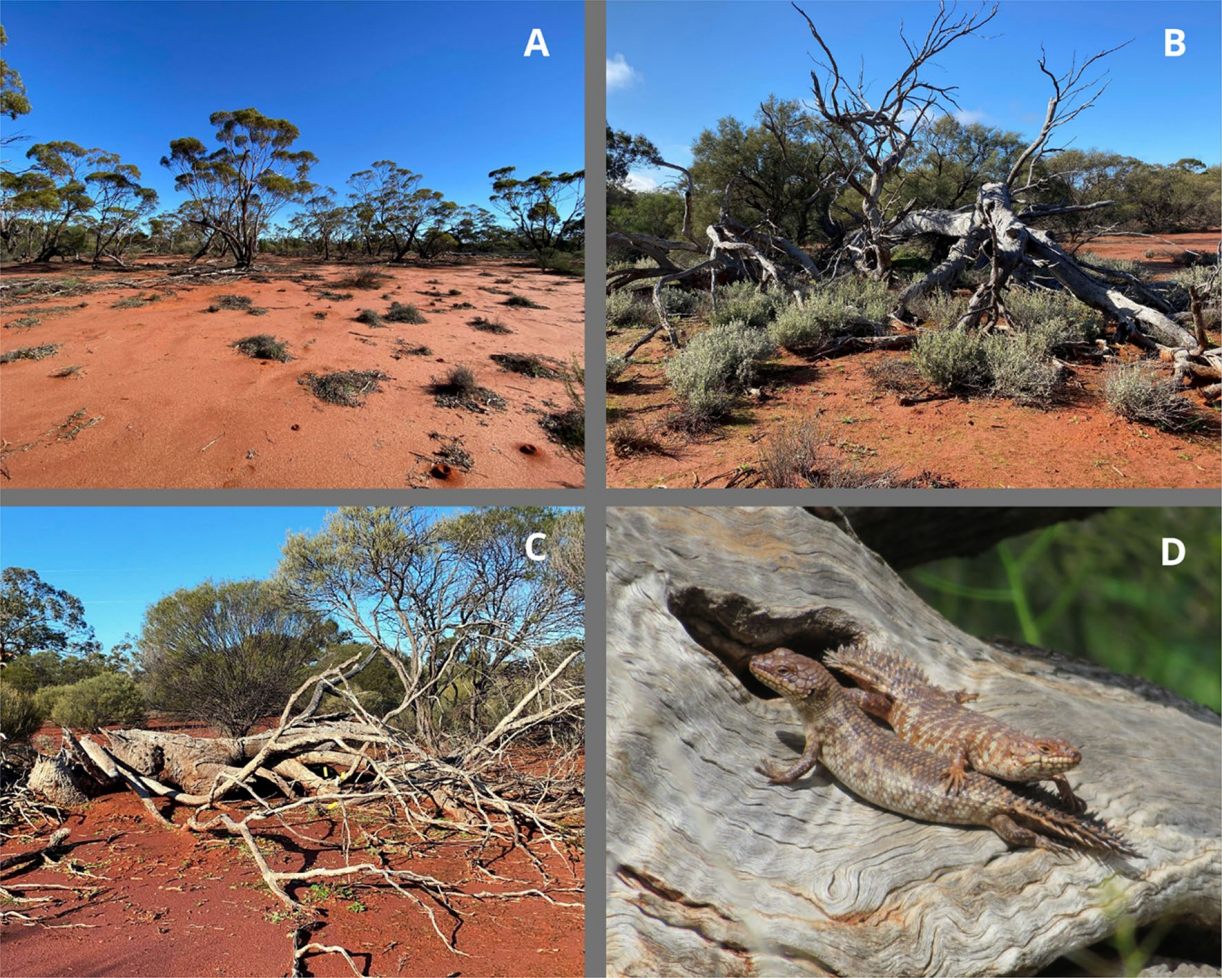
Typical habitat of the skinks in the Mid West region of Western Australia; (**A**) open *Eucalyptus* woodland in which log piles were sparsely distributed; (**B** and **C**) examples of log piles inhabited by skink colonies; and (**D**) juvenile skinks basking by one of the hollows of an occupied log pile. Photos: H Bradley.

**Figure 2:**
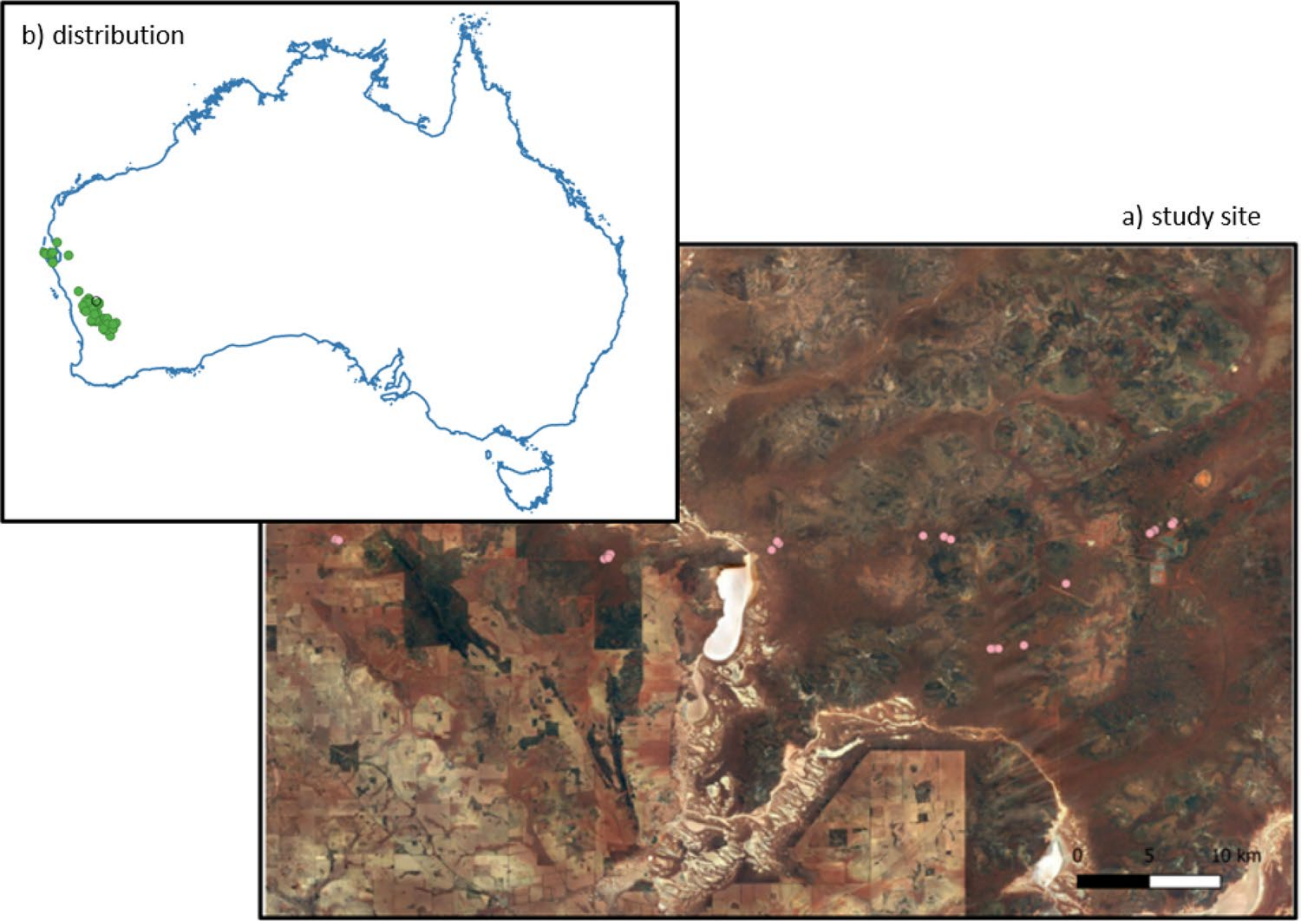
Distribution records of *Egernia stokesii badia* (orange) according to records maintained by the Atlas of Living Australia (https://www.ala.org.au/, accessed 16 December2021), and the location of the study site (black), with different LiDAR scanning locations (pink).

The study area is located approximately 450 km northeast of Perth, Western Australia, on a mining tenement in the Mid West region (29°10′54"S, 116°32′55"E). The site occurs in a semi-arid region within the distribution of the skinks and comprises mainly open eucalypt woodland on loam or clayey loam flats, predominantly York Gum (*Eucalyptus loxophleba*) over a sparse understorey^53,54^ (Fig. 1A). Log piles inhabited by the skinks (determined from previous fauna surveys) were randomly selected for study (Fig. 1B, C & D) although, due to site access limitations, log pile selection was restricted to within 1000 m of a 55 km access track (Fig. 2).

### LiDAR technology

All research and animal observational experiments were carried out and approved by the Animal Ethics Office of Curtin University (ARE2018-28) and conformed with all relevant guidelines and regulations. Scans were taken during the end of the austral winter and beginning of spring, to capture the peak abundance of annual plants. A total of 39 log piles (22 inhabited and 17 uninhabited), was scanned and three-dimensional (3D) point clouds generated for quantification of the 14 structural characteristics. The laser scanning data for this investigation were collected using a terrestrial LiDAR scanner, the Maptek I-Site 8800 (Maptek, Adelaide, Australia), which has a resolution of 10 mm^46^ and a range of up to 2000 m^55^. The LiDAR scanner was set up using a tripod on a standard tribrach mount, and a marked post was installed at each log pile site so that the scanner could be manually aligned to the top of the post using the survey alignment telescope for each scan. The scanner was placed in three to five positions around each log pile, depending on how large the log pile was, to create overlapping scans for development of a full 360-degree view of target log piles. Scan positions were targeted to ensure scanner positioning maximised capture of internal log structure within hollows. The LiDAR system position was coupled with a differential GPS system so that points were recorded with an xyz coordinate^56^.

### Data processing and analysis

**Figure 3:**
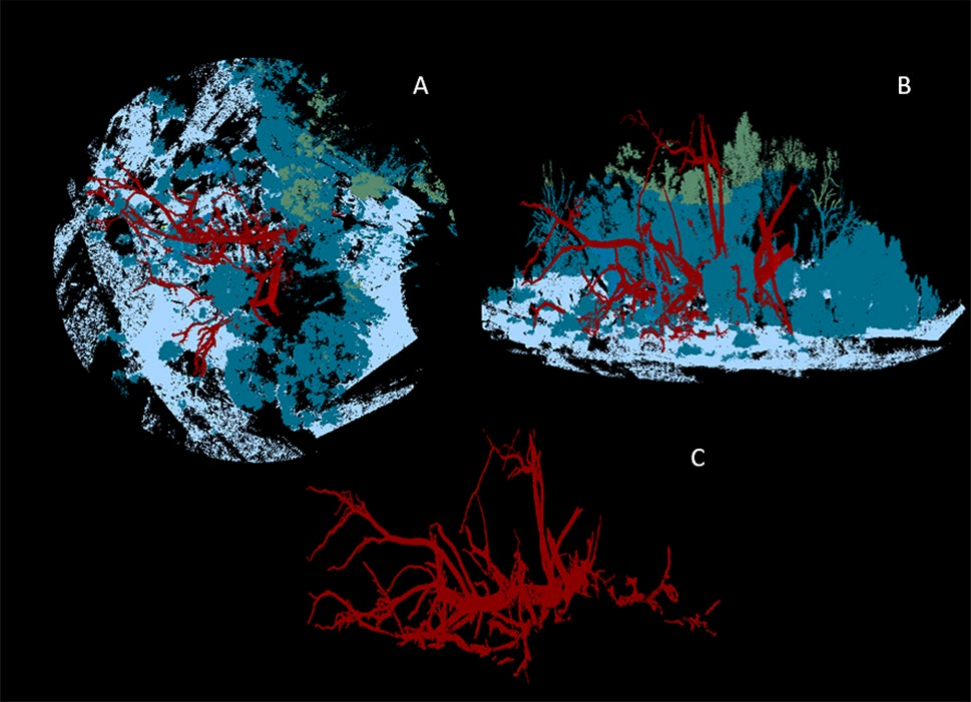
Example outputs of LiDAR scans; (**A** and **B**) the point cloud restricted within a ten-metre radius of the central log pile including the canopy cover (green), mid-storey cover (dark blue), and understorey (pale blue); and (**C**) the isolated log pile (red) from within the ten-metre radius point cloud.

Consecutive scans of each log pile were merged into a single point cloud oriented using known GPS coordinates^57^. Point clouds were then processed using the I-Site Studio software package on Maptek v5 Point Studio. High-resolution digital images taken with each scan were ‘draped’ over each point cloud to produce a 3D digital terrain model (DTM) of each log pile scene^57^. Only landscape features within a 10 m radius of each log pile were included and the model then divided into three sections: above 2 m (canopy cover), between 30 cm and 2 m (mid-storey cover), and below 30 cm (understorey; Fig. 3A & B).

Surface area along a single plane was calculated using a topographical model post-filtering for the canopy cover, mid-storey cover, and understorey layers. Manual filtering out of logs, trunks and branches prevented overestimation of vegetation cover. The processing software CloudCompare (version 2.12, 2021 retrieved from http://www.cloudcompare.org/) was used to develop system learning to isolate ‘bare ground’ versus ‘vegetation’ for understorey estimates.

Point clouds of each site were filtered to isolate each log pile system (Fig. 3C), and the physical characteristics of each log pile were measured discretely: (i) maximum canopy height, (ii) number of logs, (iii) length of log system, (iv) number of branches above and below/adjacent to the main log, (v) log structure height, (vi) diameter of widest hollow, (vii) the presence of overhanging vegetation, (viii) the position of the log pile (majority resting on ground or raised), (ix) orientation of the log pile, and (x) the diameter of the widest section of log (Table S1).

Analyses were conducted in the *R 4.04* statistical environment^58^. To determine whether the presence or absence of skinks within log piles (binary dependent variable) was predicted by log pile characteristics, multiple logistic regression models (sLRM) with continuous predictor variables (number of high branches, number of low/adjacent branches, orientation, canopy cover, mid-storey cover, understorey, number of logs, length of log system, height of log system, canopy maximum height, diameter of the widest section of log, diameter of widest hollow entrance, presence of overhanging canopy, presence of logs raised above the ground) were fitted. Missing values were replaced with the global mean of each appropriate parameter. The “dredge” function from the “MuMIn” package^59^ was used to apply a drop one protocol, to retain a candidate set of LRMs with a lower AICc (Akaike’s Information Criterion corrected for small sample sizes) than the global model. From that candidate set of models we selected the models with the lowest AICc (≤ ∆2 of the lowest AICc) and calculated the weight (ωi) of each model, which is the probability of that model being the best model. To assess the importance of individual variables we summed the weights of all models containing each variable and considered all variables with a summed model weight > 0.4 to be well supported^60^.

## Results

**Figure 4:**
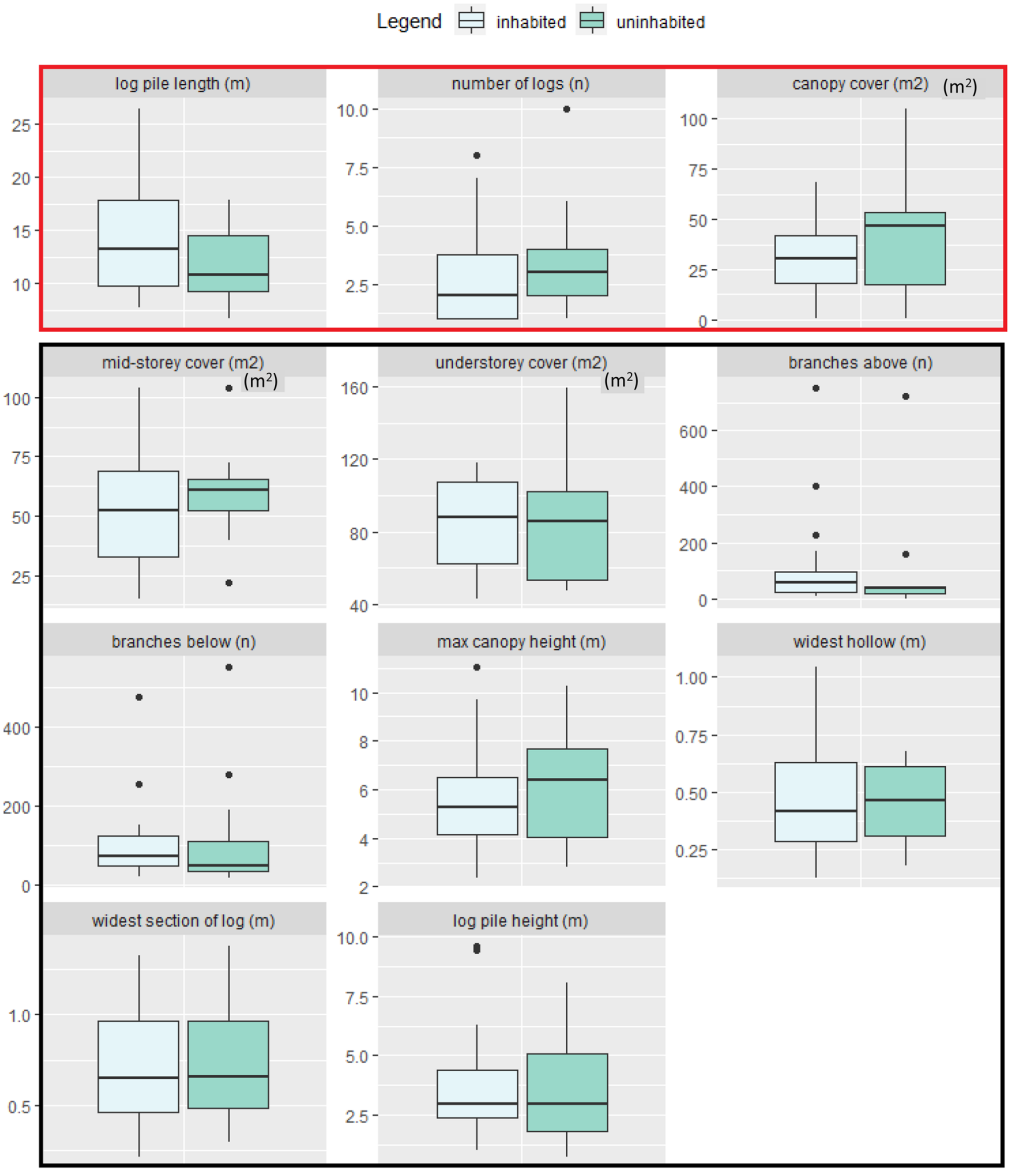
Boxplots showing the average log pile characteristics at both inhabited and uninhabited log piles. Thick horizontal lines indicate the median, boxes represent the 2nd and 3rd quartiles, and whiskers represent the 1st and 4th quartiles. Individual points represent outliers. Variables well supported to influence log pile occupancy (log pile length, number of logs and canopy cover) are bordered in red.

**Figure 5:**
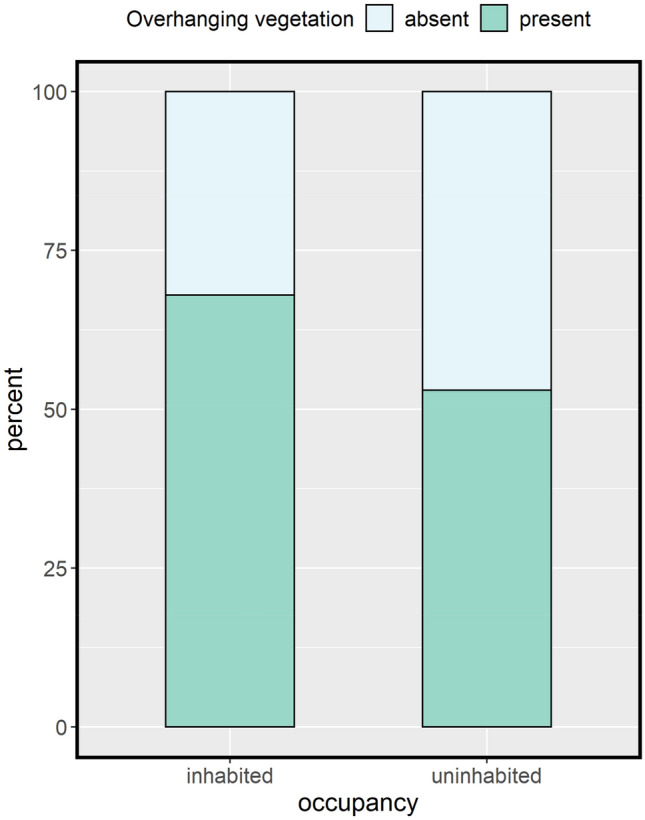
Stacked bar chart showing the differing percentage of inhabited and uninhabited log piles with vegetation overhanging the log pile (green) and with no vegetation overhanging the log pile (blue).

Compared with uninhabited log piles, inhabited log piles were generally taller with smaller entrance hollows and a wider main log, had more high-hanging branches, fewer low-hanging branches, more mid- and understorey cover, lower maximum canopy height (Fig. 4), most often faced in a SE direction (36%), and had some or most of the log pile raised off the ground (81%). The most parsimonious LRM indicated that log pile occupation by skinks was significantly predicted by increasing length of log piles (∑ω_i_ = 0.9), decreasing number of logs per pile (∑ω_i_ = 0.70), reduced canopy cover (∑ω_i_ = 0.76; Fig. 4), and the presence of overhanging vegetation (∑ω_i_ = 0.69; Fig. 5; Table S1).

## Discussion

### Microhabitat selectivity

The novel use of LiDAR to study microhabitat provided a high accuracy and resolution of structural characteristics otherwise unattainable by traditional techniques and determined four log pile characteristics to significantly influence skink presence. The most significant variable was length of the log pile, with skinks more commonly occupying longer logs. One possible reason for this trend is that taller trees may be older^61^, and larger and older trees are more likely to have hollows usable by fauna^62,63^. Tall trees (and the log piles they become) are also more likely to house a mixture of different sized cavities and hollow branches, and greater refuge options for skinks^64,65^. Therefore, before log decomposition can contribute to the production of further hollows, log piles from taller trees begin with more crevice/hollow options. Longer log piles are also likely to have the space for segregation between members of the skink colony. Due to the size variability between juveniles, adults, and gravid females, a range of crevice options is more likely to support an entire colony’s requirements^66^, as well as greater structural complexity to allow for social separation and predator refuge^67^. Other reptiles also non-randomly select trees with more branches and hollows, which is predicted to provide increased opportunity for behavioural thermoregulation^68,69^.

Skink occupation was also found to be linked with log pile composition. However, while occupancy was significantly linked with log piles averaging three logs, this was also the average for unoccupied log piles. This is likely a result of how log piles form within the landscape. The open eucalypt woodland habitat contains sparsely distributed trees or stands of trees^53,54^ which form isolated ‘habitat islands’^67^ when single or a few trees fall and decay to become log piles. The ‘logs’ within these fallen log piles were defined as the major trunks off which branches emanate. As trees are often forked and have more than one trunk, three logs are likely the average available within the landscape, or are at least the average number to provide the structural complexity (through hollow options and associated increase in branches) to support a colony.

Skinks also generally occupied log piles with overhanging vegetation combined with reduced canopy cover, indicating that the presence of vegetation, particularly at mid-storey height, adjacent to and overhanging log piles is important. Microhabitat variability helps to facilitate behavioural thermoregulation of ectotherms, and vegetation cover at a site of long-term residence is likely to be particularly important in an arid environment where vegetation is highly scattered^70^. Microhabitats that provide complex shading have been found to increase the activity budget of other arid-dwelling lizard species during hot weather, with vegetation also acting as a temperature buffer during cooler months^70^. Presence of vegetation around log piles in arid habitats can also increase the abundance and richness of reptiles, probably due to a range of benefits including increased food availability, predator refuge, and options for behavioural thermoregulation^71–73^. The effects of cover on predation are mixed: some taxa are more susceptible to predation in habitats with less vegetative cover^74^. Similarly, many species also preferentially forage in areas of vegetation^75–77^. However, other studies show that predation can increase if cover provides perches for ambush predators^78,79^, likely why skinks preferred less vegetation cover at canopy height. Therefore, selection of log piles with overhanging vegetation either benefits both thermoregulatory capacity and refuge from predation by skinks or is a trade-off between the two.

### Management implications

Within Australia, many semi-arid and arid dwelling lizard species are uncommon, with their distribution often correlated with habitat, microhabitat, or diet specificity^80^. As inappropriate habitat selection is one of the major reasons that herpetofauna translocations often fail^81^, we predicted that microhabitat structure may limit log pile suitability for skink colonies, contributing to their limited distribution within the landscape. Our results support a degree of microhabitat selectivity by skinks, with occupation linked to log pile length, number of logs, canopy cover, and overhanging vegetation. Log pile length and composition can be easily manipulated when selecting translocation sites or introducing coarse woody debris to restoration sites. However, selection of sites with reduced canopy cover, but overhanging mid-storey, may take longer to influence through management. Biomass and vegetation complexity at the understorey and mid-storey height can be significantly reduced by introduced grazers^82,83^, retaining the canopy layer which they cannot reach. As skink habitat both in our study, and regionally occurs in areas with a long history of pastoralism and landscape degradation from grazing and mining operations^84^, restoration efforts and establishment of exclusion zones may be required to recover appropriate vegetation structure prior to any translocations into the area. In areas of mining restoration, while coarse woody debris can be introduced into the landscape, growth of surrounding vegetation cover may take time to establish^85,86^, leading to a lag-phase in the development of suitable habitat for fauna recolonisation or translocation. Pre-planning is, therefore, critical to ensure that recipient sites have suitable microhabitat characteristics to support skink colonies prior to any translocations taking place.

In addition to pre-planning and active management, we also recommend additional research be undertaken to further improve our knowledge of the ecological requirements of the western spiny-tailed skink. Our observations of occupied log piles were restricted to a short window of time and our contemporary distinction between inhabited and uninhabited log piles may not be reflective of the sites most suitable to support colonies. Habitat degradation through grazing is a major threat for the skink^45^, and the study area has a long history of pastoralism^84^. Remnant skink colonies may, therefore, have been increasingly prevented from dispersing to other suitable uninhabited log piles by habitat degradation and fragmentation arising from grazing and mining infrastructure^87^. Increased predation pressure from introduced pests such as feral cats (*Felis catus*) may also have impacted dispersal capability across the landscape, as has been observed for other species of *Egernia* in degraded or disturbed landscapes^88,89^. Further research is recommended to understand if skinks have limited dispersal capacity within degraded landscapes, and if the non-dispersing older adults are remaining with younger adults and failing to establish new colonies. This research could also help determine if the trends in inhabited log pile characteristics observed in this study become more pronounced with a greater influence of habitat choice.

### Conclusions

The degree to which we could obtain highly accurate and finely resolved measurements of inhabited and uninhabited microhabitats was essential to our capacity to differentiate between the two, and the application of LiDAR made this possible in a way that conventional measurements would not have. Overall, such detailed characterisation of microhabitat structure will provide important insight into the management of a cryptic, endangered skink, such as selecting appropriate sites for translocation. Guiding translocations is, however, only one aspect of wildlife management and species recovery to which an understanding of microhabitat preferences is central. We suggest that other applications might include designing restoration landscapes to facilitate skink colony return, increasing the targeted nature of monitoring surveys, highlighting key areas within their broader habitat range for protection, and indicating areas for targeted invasive predator control. The novel application of terrestrial LiDAR for microhabitat characterisation will be a cost-effective, accurate tool with far-reaching applications in the future study of ecological systems around the world, such as the assessment of other complex microhabitat structures (e.g., roosting structures for bat species including the orange leaf-nosed bat^47^ or nesting sites of endangered parrots such as the Swift Parrot^49^) to better understand sites for protection, translocation, or replication in restoration and other threatened species management.

## Supplementary Information

Supplementary Information.

## Data Availability

The datasets generated during and/or analysed during the current study are available from the corresponding author on reasonable request.
