# Supplementary material for: Revealing microhabitat requirements of an endangered specialist lizard with LiDAR
Source: Sci Rep. 2022 Mar 25;12:5193. doi: 10.1038/s41598-022-08524-2 (PMC8956745; doi:10.1038/s41598-022-08524-2)
Supplement: Supplementary file 1 — Supplementary Information. [file 41598_2022_8524_MOESM1_ESM.docx]

**11. Supplementary information**

**Table S1.** Results of the best subset modelling of the log pile structural data showing all well-supported models (ω_i_ > 0.1) describing the relationship (whether +ve or –ve) between log pile occupancy and structural variables, plus ΔAIC_c_, ω_i_, and Nagelkerke’s R^2^ values of that model are shown. Nagelkerke’s R^2^ values represent the amount of variation explained by the model.

| **Structural variables** | **ΔAIC_c_** | **ω_i_** | **Nagelkerke’s R^2^** |
| --- | --- | --- | --- |
| Canopy cover (-ve)  Length of log system (+ve)  Number of logs (-ve)  Presence of overhanging vegetation (+ve) | 48.43 | 0.18 | 0.469 |
